# Series Arc Fault Detection Based on Multimodal Feature Fusion

**DOI:** 10.3390/s23177646

**Published:** 2023-09-04

**Authors:** Na Qu, Wenlong Wei, Congqiang Hu

**Affiliations:** School of Safety Engineering, Shenyang Aerospace University, Shenyang 110136, China; w7wwl995@163.com (W.W.); hu_congqiang@163.com (C.H.)

**Keywords:** series arc fault, multimodal feature fusion, machine learning, one-dimensional convolutional network, deep residual shrinkage network

## Abstract

In low-voltage distribution systems, the load types are complex, so traditional detection methods cannot effectively identify series arc faults. To address this problem, this paper proposes an arc fault detection method based on multimodal feature fusion. Firstly, the different mode features of the current signal are extracted by mathematical statistics, Fourier transform, wavelet packet transform, and continuous wavelet transform. The different modal features include one-dimensional features, such as time-domain features, frequency-domain features, and wavelet packet energy features, and two-dimensional features of time-spectrum images. Secondly, the extracted features are preprocessed and prioritized for importance based on different machine learning algorithms to improve the feature data quality. The features of higher importance are input into an arc fault detection model. Finally, an arc fault detection model is constructed based on a one-dimensional convolutional network and a deep residual shrinkage network to achieve high accuracy. The proposed detection method has higher detection accuracy and better performance compared with the arc fault detection method based on single-mode features.

## 1. Introduction

When a series arc fault occurs, the current value is often less than the threshold of the circuit breaker. When the arc burns, its temperature can reach thousands of degrees, and it is not easily extinguished. While the voltage is 20 V, the arc can still maintain a continuous and stable combustion. Statistical analysis shows that the proportion of electric fires caused by arc faults is up to 50%. Therefore, arc fault detection is important to ensure electrical safety [1,2].

Currently, extensive research on series arc fault detection is being conducted by scholars from various perspectives. The research can be categorized into three main areas. Firstly, researchers focus on arc fault detection by analyzing its physical characteristics. Sensor technology is employed to detect indicators such as arc light, arc sound, temperature, and electromagnetic radiation, enabling the determination of whether an arc fault has occurred [3,4,5,6]. Secondly, simulation research is employed based on mathematical models of arcs. Notable models include Cassie [7,8], Mayr [9,10], Schavemaker [11,12], and Habedank [13]. By utilizing computer simulation technology instead of conducting complex arc experiments, significant cost savings can be achieved. Thirdly, researchers investigate the detection and analysis of current signals under normal operation and arc fault conditions. This approach involves analyzing the time–frequency features of the current signal, extracting relevant features, and utilizing artificial intelligence algorithms for detection. It is currently one of the most important research directions in arc fault detection [14,15,16,17]. In addition, some researchers have started to explore the fusion of different current signal features to obtain more informative features and improve the discrimination of feature data [18,19,20]. However, most of these feature fusion methods focus on fusing features within the same modality, overlooking the complementary nature of information between different modalities. Additionally, their feature fusion approaches primarily rely on direct concatenation fusion of low-level features, resulting in fused features that contain amounts of noise and redundant information, thus impacting the accuracy of arc fault detection.

To address the above problems, this paper proposes an arc fault detection method based on multimodal feature fusion. It utilizes the complementary nature of different modalities’ information to achieve accurate detection of series arc faults. Wang [21] reduces the sampling error and improves the efficiency and accuracy of a vehicle detection system by matching the coordinate relationship between the two sensors. Virk [22] uses this multimodal technique to assign appropriate weights to the features obtained in multiple domains, thus improving the detection accuracy. Liang [23] improves the accuracy of false information detection by integrating text features and visual features and taking low-dimensional features as joint features of multiple modes.

The main contributions of this paper are as follows:

(1) The current signal features of normal operation and arc fault under different load conditions are analyzed.

(2) According to the analysis results of the current signal, methods such as mathematical statistics, Fourier transform, wavelet packet transform, and continuous wavelet transform are used to extract the time-domain statistical features, frequency-domain harmonic factor, harmonic distortion rate features, wavelet packet energy features, and continuous wavelet transform time–frequency image features. These features all reflect current signal characteristics to a certain extent.

(3) In order to solve the problem of low detection accuracy caused by low feature quality, a feature data processing method is proposed. Random forest, gradient boosting tree, and limit gradient boosting tree are used to select one-dimensional data features. Then, grayscale and principal component analysis (PCA) are used to reconstruct the time–frequency image features to improve the feature data quality.

(4) According to the idea of ensemble learning and decision-level feature fusion, a series arc fault detection model is constructed using one-dimensional convolutional networks and deep residual shrinkage networks. This model achieves high accuracy in arc fault detection, with an average detection accuracy of 98.87%. In a set of 1000 random test samples, the detection accuracy reaches 99.30%, which demonstrates strong generalization ability.

## 2. Current Signal Analysis and Feature Extraction

### 2.1. Current Signal Analysis

**Figure 1:**
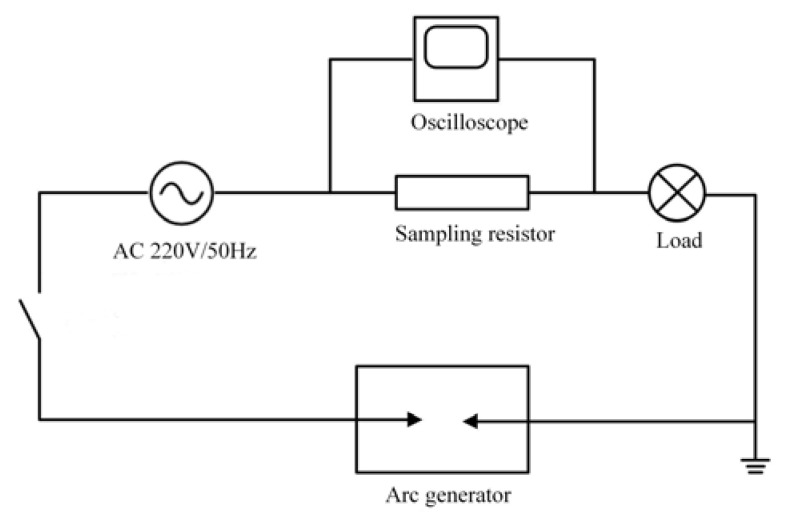
Schematic diagram of series arc fault experiment.

**Figure 2:**
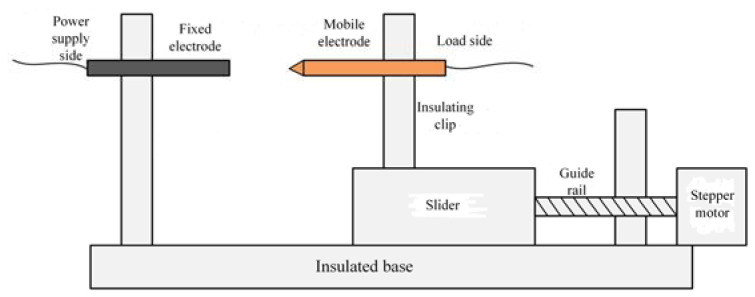
Structural schematic diagram of arc-generating device.

According to the UL1699 international standard, a series arc fault experimental platform is constructed. It mainly includes the arc fault generator, experimental loads, 220 V/50 Hz AC power supply, sampling resistors, and oscilloscopes, as shown in Figure 1 and Figure 2. The arc generator is the core of the series arc fault simulation experimental platform design. The TDS1001C-SC of Tektronix oscilloscope and the TPP0101 10× voltage probe are selected to complete the experiment. The sample interval is 4 × 10^−4^ s. The current waveform of the circuit is obtained by the sampling resistance method. The six kinds of electrical loads that are commonly used in low-voltage distribution systems are selected. As shown in Table 1, the experimental loads can be divided into two types: linear loads and nonlinear loads.

**Figure 3:**
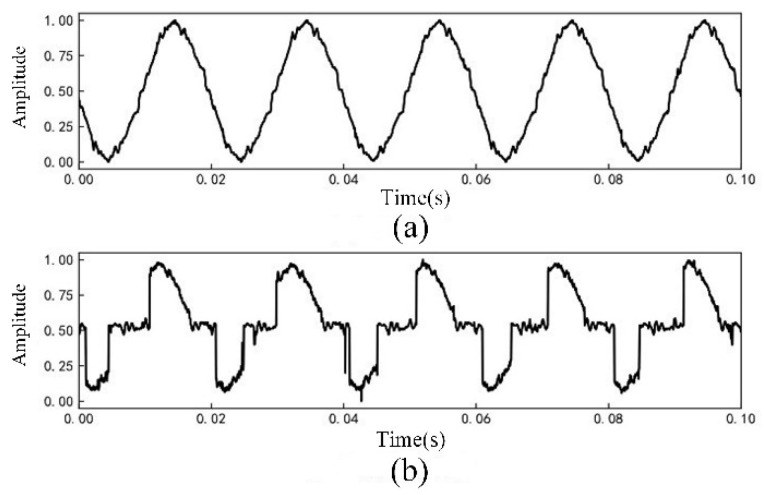
Incandescent lamp load current waveforms: (**a**) Normal current; (**b**) Arc fault current.

**Figure 4:**
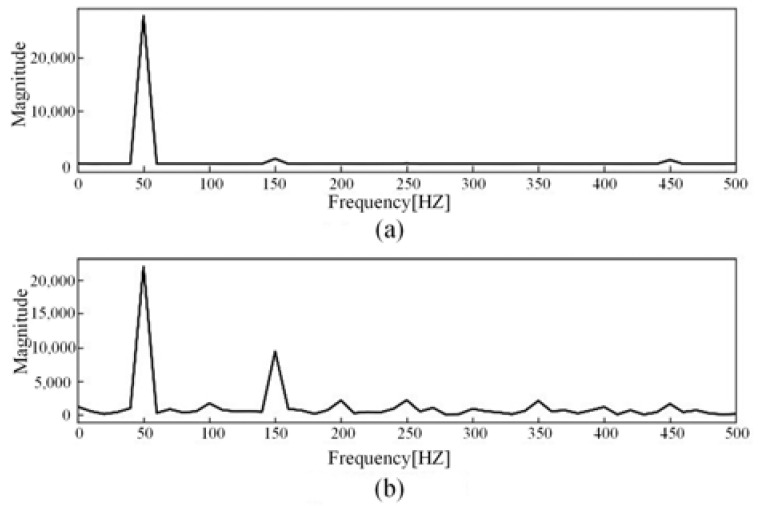
Incandescent lamp load current amplitude spectrum: (**a**) Normal current; (**b**) Arc fault current.

**Figure 5:**
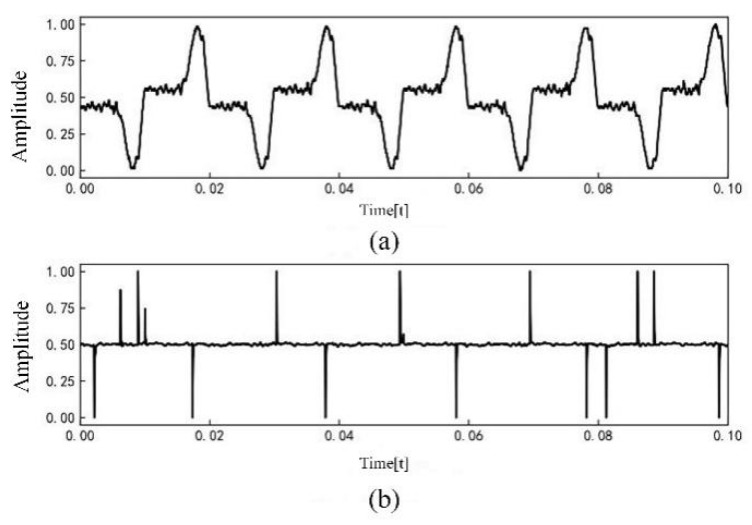
Computer load current waveforms: (**a**) Normal current; (**b**) Arc fault current.

**Figure 6:**
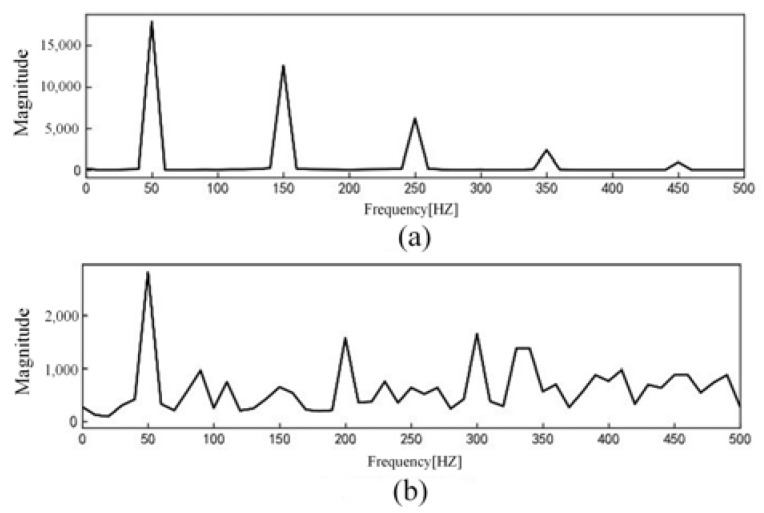
Computer load current amplitude spectrum: (**a**) Normal current; (**b**) Arc fault current.

The current signal data are normalized and further processed by Fourier transform to obtain the current amplitude spectrum. Among them, the linear load is exemplified by an incandescent lamp load. The current waveform is shown in Figure 3, and the current amplitude spectrum is shown in Figure 4. The nonlinear load is exemplified by a computer load. The current waveform is shown in Figure 5, and the current amplitude spectrum is shown in Figure 6.

The comparative analysis of current signal characteristics during normal operation and arc fault revealed the following observations. When the load is linear, the current waveform is a relatively regular sine wave during normal operation. However, when an arc fault occurs, the current waveform is distorted to a certain extent, and there is a “zero rest” phenomenon. The fault current contains some high-order harmonics. When the load is nonlinear, the current waveform of normal operation may be similar to the “zero rest” phenomenon and exhibit severe distortion. When arc fault occurs, the current contains some high-order harmonics and the odd harmonic amplitude growth is more obvious.

### 2.2. Current Signal Feature Extraction

#### 2.2.1. Time-Domain Feature Extraction

Signal analysis methods mainly include time-domain analysis, frequency-domain analysis, and time–frequency analysis. Among them, time-domain analysis is the simplest and most straightforward method. It can quickly and intuitively reflect signal features and is widely used in fields such as fault detection and optical communication. Time-domain analysis enables the extraction of various features, both dimensional and dimensionless. Dimensional features are susceptible to environmental and load influences, while dimensionless features remain unaffected by such factors, making them more suitable for capturing the current state and extracting signal features. Time-domain features such as mean value, crowd value, standard deviation, mean square value, root mean square value, skewness index, kurtosis index, peak index, waveform index, pulse index, margin index, etc., are selected.

#### 2.2.2. Frequency-Domain Feature Extraction

**Figure 7:**
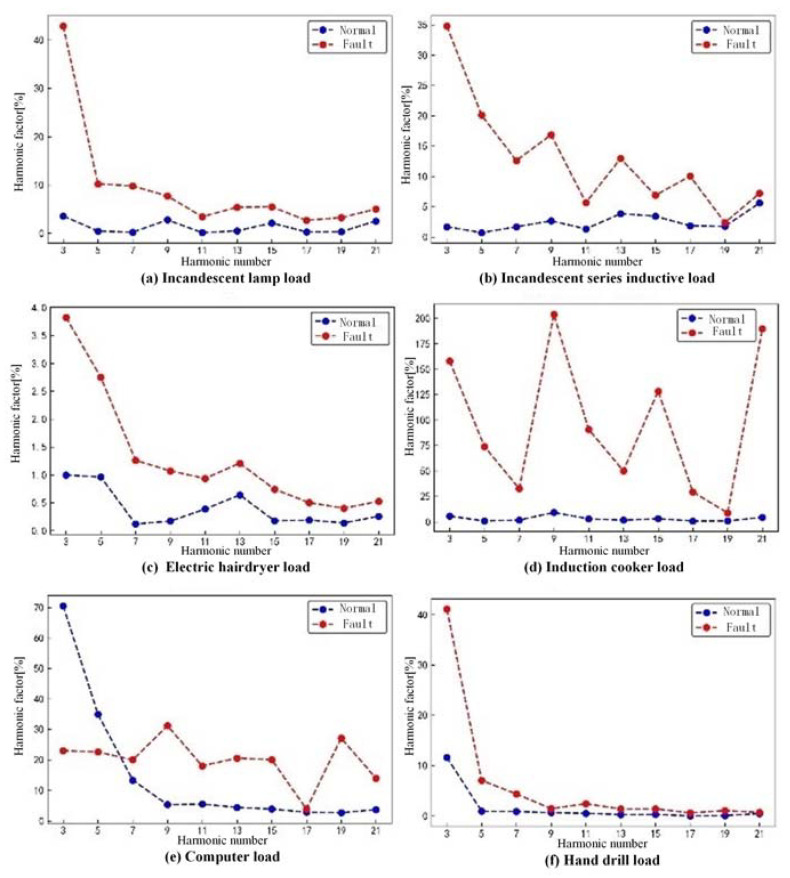
Odd harmonic factor for each load at different states.

Time-domain analysis provides a straightforward representation of signal features but lacks the ability to reveal deeper underlying information. Fourier transform can decompose signals into sine waves of different frequencies, thereby processing complex signals and extracting more feature information. During normal operation, the current amplitude spectrum is mainly concentrated at the fundamental frequency of 50 HZ. When a series arc fault occurs, the amplitude of odd harmonics increases significantly compared with that under normal operation conditions, and the increase in odd harmonic amplitude is not affected by the load type. Considering the influence of load on harmonic amplitude, a harmonic factor is introduced to represent the change in harmonic content in the current signal. The calculation principle is shown in Formula (1).

(1) $$P_{k}=\frac{H_{k}}{H_{1}}\begin{array}{cc} & k=2,3,\ldots ,n \end{array}$$

where ***P_k_*** is the ***k*th** harmonic factor, ***H_k_*** is the ***k*th** harmonic amplitude, ***H*_1_** is the fundamental amplitude, ***k*** is the number of harmonics, and ***n*** is the maximum number of harmonics. In this paper, the odd harmonic amplitude variation is studied, and *k* is taken as an odd number. From the spectrum analysis, it is observed that the amplitude of harmonics decreases as the harmonic order increases. To avoid the issue of very small amplitudes for high-order harmonics, the first ten odd harmonic factors are selected as feature indicators for detecting series arc faults. Figure 7 illustrates the distinct differences in odd harmonic factors between normal operation and arc fault conditions, making them valuable features for detecting series arc faults. In addition, when the arc fault occurs, the fault current waveform appears with different degrees of distortion. Therefore, this paper calculates the total harmonic distortion rate of the current signal through the Fourier transform, which is used to describe the degree of distortion of the current waveform relative to the sine wave, abbreviated as *THD*. The calculation principle is shown in Formula (2).

(2) $$THD=\sqrt{\sum_{k=2}^{H}(\frac{G_{k}}{G_{1}}{)}^{2}}$$

where *THD* is the total harmonic distortion rate, *G_k_* is the *k*th harmonic rms value, *G*_1_ is the fundamental *rms* value, *k* is the number of harmonics, and *H* is the specific order, generally taken as 2–39. The amplitude value of high-order harmonic waves is very small, and its impact on the total harmonic distortion rate can be ignored. To reduce the computational complexity, *H* is taken as 20. The total harmonic distortion rate can be used to detect whether an arc fault occurs.

#### 2.2.3. Wavelet Packet Energy Feature Extraction

According to the amplitude spectrum of the current signal, when an arc fault occurs, the harmonic content changes significantly. This change is not only reflected in the integer-order harmonics, but also in the interharmonics. More accurate analysis methods are needed to analyze the current signal. Wavelet packet analysis enables better time–frequency localization analysis of signals containing a large amount of low- and high-frequency information. Therefore, this study uses wavelet packet transform to process current signals and achieve interharmonics energy calculation. Wavelet packet transform is an extension of wavelet transform. Orthogonal wavelet packet bases are generated from a standard Orthogonalization scale parameter through a two-scale difference equation [21,22]. The calculation of the orthogonal wavelet packet bases is shown in Equation (3), and the biorthogonal difference equation is represented by Equation (4).

(3) $$w_{n,j,k}(t)=2^{-j/2}w_{n}(2^{-j}t-k,n\in Z/Z^{-1},j,k\in Z)$$

(4) $$\left\{\begin{array}{c} w_{2n}(t)=\sqrt{2}\sum_{K\in Z}h_{k}w_{n}(2t-k)\\ w_{2n+1}(t)=\sqrt{2}\sum_{K\in Z}g_{k}w_{n}(2t-k) \end{array}\right.$$

where *n, j*, and *k* represent the positions of wavelet packets in the grading, *h_k_* is the low-pass filter, and *g_k_* is the high-pass filter. The wavelet packet decomposition coefficients are obtained from the projection of the signal *S(t)* in the orthogonal wavelet basis space. The calculation is shown in Equation (5).

(5) $$c_{n,j,k}=\int_{-\infty }^{+\infty }S(t)w_{n,j,k}(t)dt$$

When the wavelet packet basis function is a set of orthogonal bases, the wavelet packet transform has the property of energy conservation. The energy of the wavelet packet at a single scale is the sum of squares of the wavelet packet coefficients at that scale [23]. The calculation can be expressed as shown in Equation (6).

(6) $$E_{j}=\sum_{k}c_{n,j,k}^{2}$$

In the process of wavelet packet decomposition, it is necessary to select an appropriate wavelet function based on the signal characteristics. The Daubechies series wavelet is favorable for processing sudden signals such as arc faults due to its orthogonality, tight support of time–frequency, high regularity, and Mallat fast algorithm. In the process of current signal decomposition, too many layers of decomposition will lead to an increase in the amount of calculation. With the increase in the number of layers, the effective information will also be reduced, resulting in data redundancy, affecting the accuracy and efficiency of detection, while too few layers of decomposition will lead to incomplete features. After comprehensive consideration, five-layer wavelet decomposition is performed to extract the energy features of the wavelet packet.

**Figure 8:**
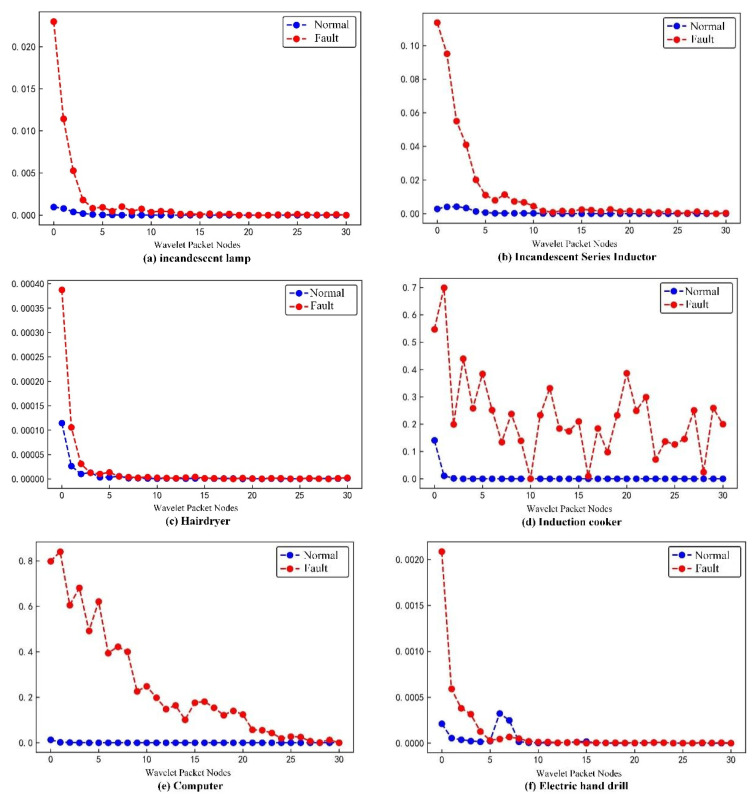
Wavelet packet energy for each load at different states.

As shown in Figure 8, the wavelet packet energy of each node is normalized to facilitate the observation of its variations. It can be seen that there are differences in the wavelet packet energy of the same node under different load conditions, and the differences decrease gradually with an increasing number of wavelet packet nodes. Therefore, the extracted wavelet packet energy feature can be used to detect whether an arc fault occurs.

#### 2.2.4. Continuous Wavelet Transform Image Features

In actual arc fault current signals, there are many nonstationary and random components. It is necessary to use wavelet analysis methods to extract features such as transients, singularities, and mutations from the signal. In this paper, continuous wavelet transform is employed as a means of extracting arc fault current features. The wavelet detail coefficients at each level are extracted as feature information for detecting arc faults and transformed into wavelet time–frequency image features. Compared to one-dimensional features, time–frequency image wavelet features are two-dimensional data matrices that carry more information. The basic principle is as follows [24,25].

Let $\psi (t)\in L^{2}(R)$, $L^{2}(R)$ represent a real number space of square integrable such that the Fourier transform satisfies the condition.

(7) $$C_{\psi }=\int_{-\infty }^{+\infty }|\psi^{\prime }(\omega ){|}^{2}\frac{d\omega }{\left|\omega \right|}<\infty$$

where $C_{\psi }$ is the permittivity condition, and ψ(t) is the basis wavelet or wavelet mother function. ψ(t) is scaled and translated to obtain the following function.

(8) $$\psi_{a,b}(t)=\frac{1}{\sqrt{\left|a\right|}}\psi (\frac{t-b}{a})$$

where $\psi_{a,b}(t)$ is the continuous wavelet basis function, *a* is the scale parameter, *b* is the translation parameter, and a,b∈R(a≠0). For an arbitrary function $f(t)\in L^{2}(R)$ and ψ(t) represent a wavelet basis function, and the result of the continuous wavelet transform is as

(9) $$W_{f}(a,b)=|a{|}^{-\frac{1}{2}}\int_{-\infty }^{+\infty }f(t)\bar{\psi (\frac{t-b}{a})}dt,\;a\neq 0$$

where $W_{f}(a,b)$ is the wavelet transform of the function *f*(*t*) with respect to the function $\psi_{a,b}(t)$, and $\bar{\psi (t)}$ is the conjugate function $\psi_{a,b}(t)$.

In extracting time–frequency features using continuous wavelet transform, the selection of wavelet basis function and scaling parameter “*a*” is crucial. This study chooses the *db*5 wavelet function as the mother wavelet for continuous wavelet decomposition. In this paper, all the loads are tested, and the appropriate scaling scale is selected. The scaling parameters for the continuous wavelet transform of each load are presented in Table 2.

**Figure 9:**
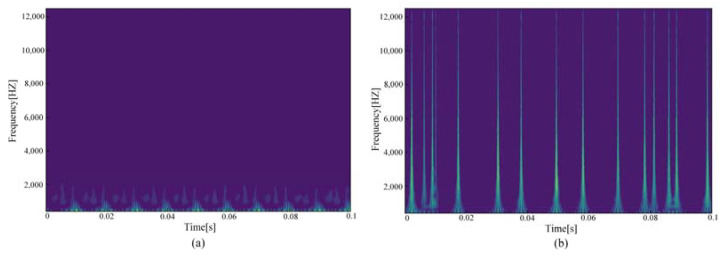
Computer load time–frequency image feature: (**a**) Normal current; (**b**) Arc fault current.

Taking the computer load current as an example, the time–frequency image features obtained from the continuous wavelet transform are shown in Figure 9. The nonstationary and random components in the current signal are reflected in the continuous wavelet transform time–frequency image. There are obvious differences between time–frequency images of normal operation and arc faults, which can be used as an important feature to detect arc faults.

## 3. Arc Fault Feature Data Processing

### 3.1. Feature Selection

**Figure 10:**
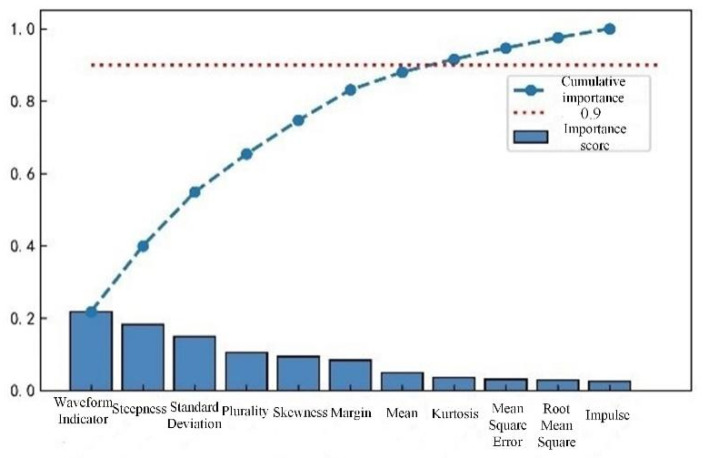
Cumulative importance of time-domain features.

**Figure 11:**
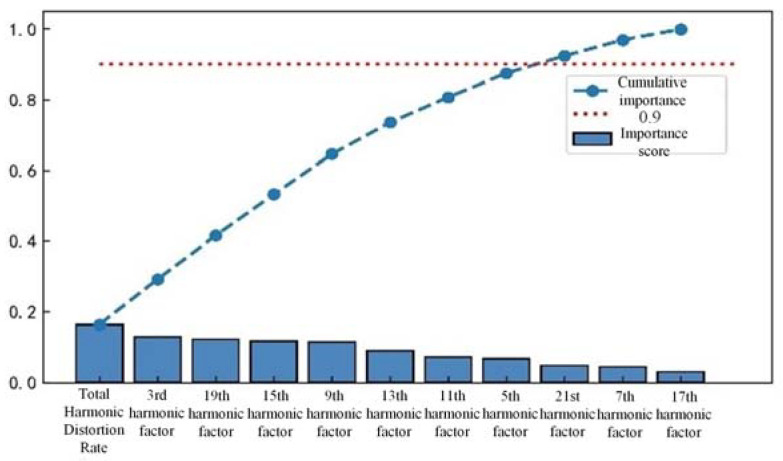
Frequency-domain cumulative importance of features.

**Figure 12:**
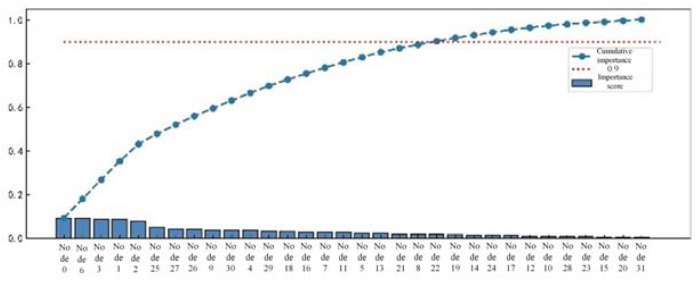
Importance ranking of integrated wavelet packet energy features.

There may be redundant and invalid features in the preliminary feature extraction, which affects the efficiency and accuracy of series arc fault detection. Three different tree algorithms are used to rank the importance of one-dimensional features, such as time-domain features, frequency-domain features, and wavelet packet energy features. Feature selection is made according to the ranking results, as shown in Figure 10, Figure 11 and Figure 12.

Based on the sorting results, retaining the top 90% of the cumulative importance features not only avoids excessive loss of feature information, but also ensures detection efficiency. In time-domain features, features with lower importance, such as pulse indicators, root mean square values, and mean square values, are removed while retaining the remaining eight features. In the frequency-domain features, the 17th and 21st harmonic factors are removed, while the remaining 9 features are retained. In the energy features of wavelet packets, 10 features with lower importance are removed, while 22 features with higher importance are retained.

### 3.2. Time–Frequency Image Grayscale

**Figure 13:**
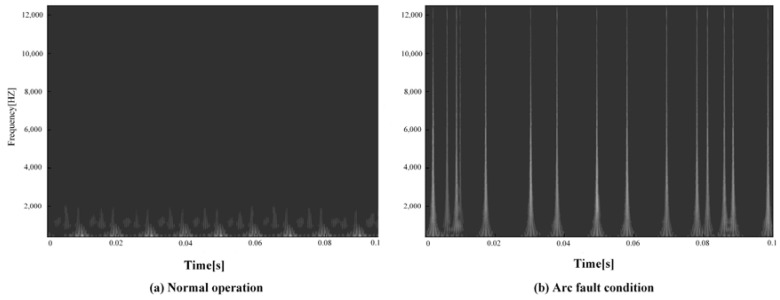
Grayization of computer load time–frequency image.

Each pixel’s color is determined by three components, i.e., red, Green, and blue. Each component ranges from 0 to 255, and a pixel can have more than 16 million color variations. The gray-level processing of a feature image can change the three-channel into a single-channel, which is beneficial to reduce the computation. There are three kinds of image grayscale algorithms: maximum-value method, average-value method, and weighted average-value method. The maximum method generates images with high brightness. The average method generates softer images. The images generated by the weighted average method are most easily recognized by computers. This paper uses the weighted average method, and the calculation principle is shown in Equation (10). Taking the computer load current as an example, the grayscale time–frequency feature images are shown in Figure 13.

(10) $$\mathrm{Gray}(i,j)=w_{R}R+w_{G}G+w_{B}B$$

where *i* and *j* are the horizontal and vertical coordinates of the pixel points, respectively; Gray(i,j) is the grayscale function; $w_{R}$, $w_{G}$, and $w_{B}$ are the weights of *R*, *G*, and *B*, respectively; and generally, when $w_{R}=0.299$, $w_{G}=0.587$, and $w_{B}=0.114$, the grayscale effect is the best.

### 3.3. Time–Frequency Image Feature Reconstruction

The image structure after grayscale processing is (128, 128, 1), encompassing 16,384 feature values. It can be found that there are a large number of highly correlated and invalid features by analyzing the grayscale image. There are fewer effective feature values that can identify the load type and state. In response to this issue, this paper uses principal component analysis (PCA) to reconstruct time–frequency image features. Assuming there are *N* images, *X_i_* is the column vector of the *i*-th image, and *X* is the combination matrix of *N* images. The overall covariance matrix is given by

(11) $$\mu =\frac{1}{N}\sum_{i=0}^{N-1}X_{i}$$

(12) $$C=\frac{1}{N}\sum_{i=0}^{N-1}(X_{i}-\mu )(X_{i}-\mu {)}^{T}=\frac{1}{N}XX^{T}$$

where μ is the average image vector of the sample set images, and *C* is the covariance matrix. Let the eigenvalues of the covariance matrix be $\lambda_{i}$ and the corresponding eigenvectors be $u_{i}$. The top *L* largest eigenvectors are selected to construct the projection matrix $E=(u_{0},u_{1},\cdot \cdot \cdot ,u_{L-1})$, where *L* is determined by the cumulative contribution rate α of the eigenvalues.

(13) $$\alpha \leq \frac{\sum_{i=0}^{L-1}\lambda_{i}}{\sum_{i=0}^{mn-1}\lambda_{i}}$$

If α=0.95 is taken while preserving the internal information of the original feature vector as much as possible, then L≥216. Therefore, this article takes L=256 to reconstruct the time–frequency image features.

## 4. Series Arc Fault Detection Algorithm

This paper proposes a series arc fault detection method based on multimodal feature fusion. The different arc fault detection submodel is designed and built according to the different mode feature. The submodels are integrated, and the decision results are fused to form new feature vectors. Aiming at one-dimensional features such as time-domain features, frequency-domain features, and wavelet packet energy features, a one-dimensional convolutional neural network is designed. It includes two convolution layers, three batch normalization layers, one maximum pooling layer, one flat layer, and two full connection layers. After the convolutional layer and maximum pooling layer, the feature data are normalized in batches to avoid gradient vanishing and explosion caused by data changes. The activation function of the convolution layer is ReLU. The output layer is the full connection layer, and the activation function is SoftMax.

**Figure 14:**
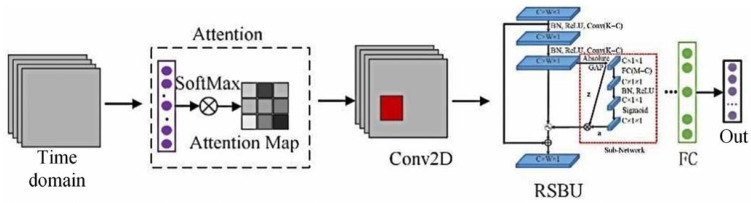
Attention-DRSN arc fault detection model.

**Figure 15:**
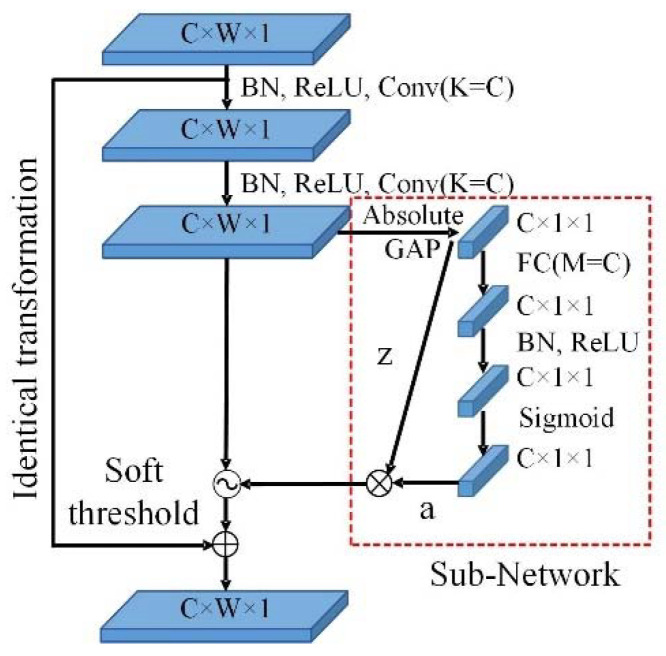
Network structure of residual shrinkage layer.

The arc fault detection model of the deep residual systolic network is designed for the time–frequency image features. This architecture includes an attention layer, a convolution layer, a residual systolic layer, three batch normalization layers, two maximum pooling layers, one average pooling layer, and two fully connected layers. The attention layer uses the SoftMax activation function, and the convolution layer uses the ReLU activation function. The Attention-DRSN arc fault detection model is shown in Figure 14. The network structure of the residual systolic layer is shown in Figure 15. The automatic soft thresholding of the model is achieved by embedding a subnetwork in the residual module, which can adaptively eliminate noise and redundant information in the feature learning process [26].

An attention mechanism is a mechanism that focuses attention on key feature information. It is mainly divided into two steps: Step 1, obtaining local key information through global scanning; Step 2, enhancing effective information to suppress ineffective information. For N input messages, $X=[X_{1},X_{2},\cdots ,X_{N}]$ has:

(14) $$\alpha_{i}=\mathrm{Softmax}[s(X_{i},q)]=\frac{\exp[s(X_{i},q)]}{\sum_{j=1}^{N}\exp[s(X_{j},q)]}$$

where $\alpha_{i}$ is the attention distribution; SoftMax is the normalized exponential function; q is the query vector; and $s(X_{i},q)$ is the attention scoring function, using the dot product model $s(X_{i},q)=X_{i}^{T}q$.

Calculated from the attention distribution:

(15) $$S=\mathrm{Att}(X,q)=\sum_{i=1}^{N}\alpha_{i}X_{i}$$

where *S* is the output result, and Att(X,q) is the attention mechanism function.

The residual shrinkage layer consists of two convolution (Conv) units and a subnetwork composed of fully connected layers (FC). C is the number of channels in the feature map, *W* is the width of the feature map, l indicates the depth of the feature map, and *K* is the number of kernels in each convolutional layer. Batch normalization (BN) is utilized to normalize the data distribution and prevent parameter saturation. ReLU is used as the activation function. After taking the absolute value of feature *x*, global average pooling (GAP) is performed, and the result *z* is used as the input to the subnetwork. The sigmoid function is the output function of the subnetwork, scaling the output value to between (0, 1). Finally, the threshold is obtained by multiplying *z* with the output value a to achieve soft thresholding. The calculation principle is as follows.

(16) $$z=\left|\bar{X}\right|$$

(17) $$a_{c}=\frac{1}{1+e^{-z_{c}}}$$

(18) $$\tau_{c}=z\cdot a_{c}$$

where *X* is the output feature image from the previous convolutional layer, *z_c_* is the neuron feature in the *c*-th layer, and *ac* is the scaling parameter of the *c*-th layer. $\tau_{c}$ represents the *c*-th channel threshold of the feature image. The soft thresholding function is calculated as follows.

(19) $$y=\left\{\begin{array}{l} x-\tau ,\begin{array}{cc} \; & x>\tau \end{array}\\ 0,\begin{array}{ccc} \; & \; & -\tau \leq x\leq \tau \end{array}\\ x+\tau ,\begin{array}{cc} \; & x<\tau \end{array} \end{array}\right.$$

where *x* is the input feature, *y* is the output feature, and τ is the threshold value. The derivative is applied to the function, and the corresponding formula is presented as (19). The soft threshold function allows for setting certain interval features to 0 while retaining the valid features to enhance the map process.

(20) $$\frac{\partial y}{\partial x}=\left\{\begin{array}{l} 1,\begin{array}{cc} \; & x>\tau \end{array}\\ 0,\begin{array}{cc} \; & -\tau \leq x\leq \tau \end{array}\\ 1,\begin{array}{cc} \; & x<\tau \end{array} \end{array}\right.$$

**Figure 16:**
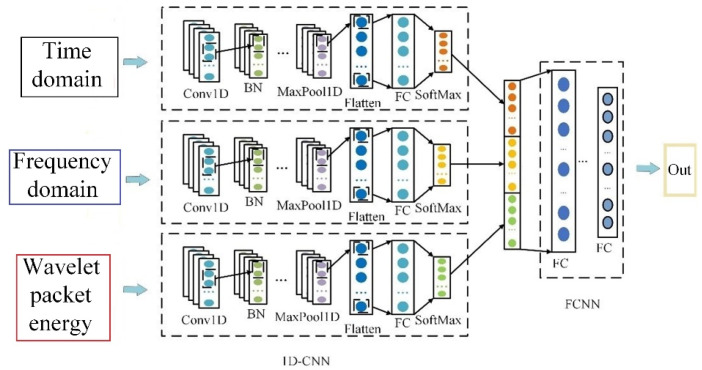
Multidomain feature fusion arc detection model.

The decision-level feature fusion layer consists of three fully connected layers. The activation function of the first two layers is ReLU, and L2 regularization is applied to these layers. The last full connection layer is output as the test result, and the activation function is SoftMax. The optimizer uses the Radam optimization algorithm to dynamically adjust parameters such as learning rate during training. Finally, the classification cross-entropy function is used as the loss function to evaluate the performance of the model. The multidomain feature fusion arc detection model is shown in Figure 16.

**Figure 17:**
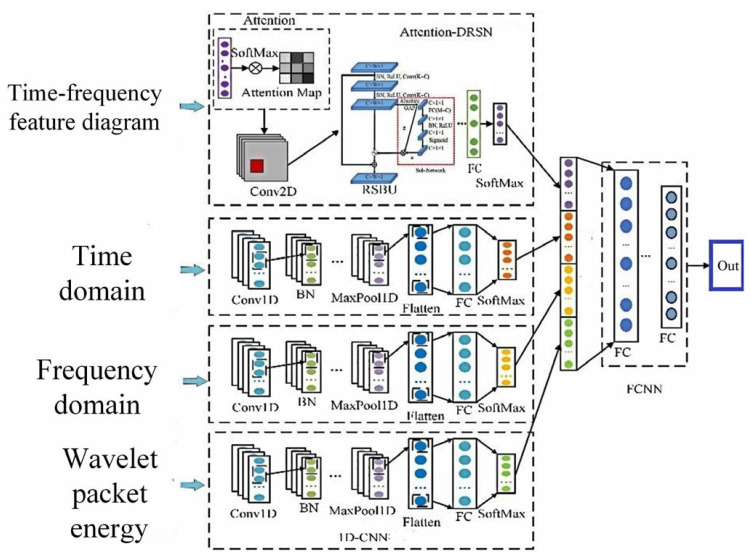
Multimodal feature fusion fault detection model.

An arc fault detection method based on multimodal feature fusion is proposed as shown in Figure 17 in order to obtain more characteristic information, increase the feature differentiation of current signal under different load states, and improve the arc fault detection accuracy.

## 5. Result

### 5.1. Result Analysis

**Figure 18:**
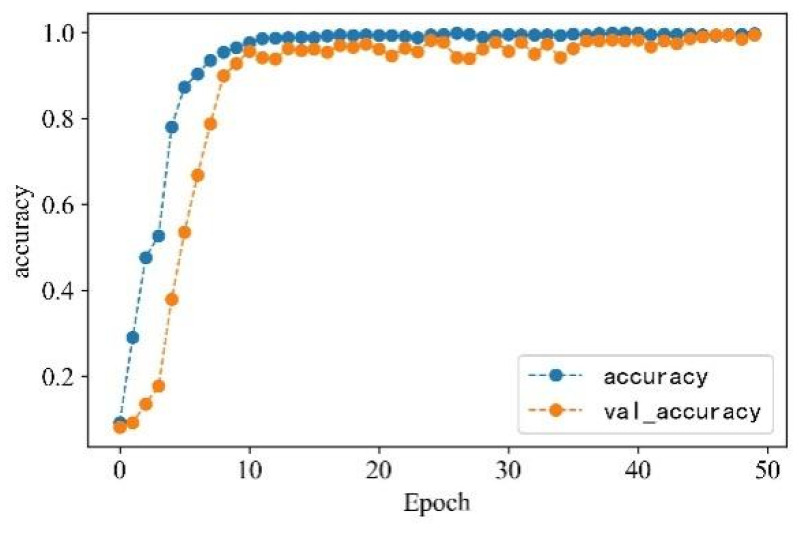
Accuracy change curve.

**Figure 19:**
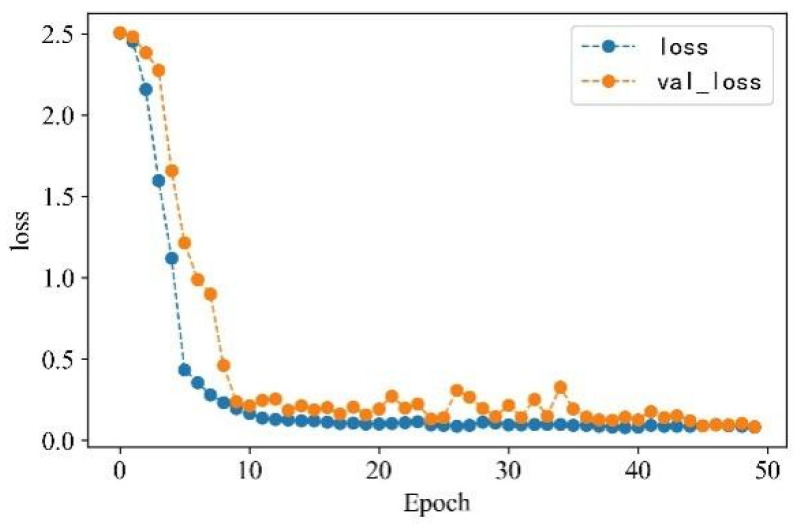
Loss value change curve.

In the serial arc fault detection experiment, the processed feature data samples are labeled, and the labels are one-hot encoded. Four feature datasets are constructed through time-domain, frequency-domain, wavelet packet energy, and time–frequency image features. A total of 530 sets of each type of feature under different loads and states are taken as detection samples. The data sets are scrambled and divided into training and validation sets in a 3:1 ratio to train and verify the model. Each epoch contains 16 data samples, and the number of iterations is set to 50. The detection accuracy is shown in Figure 18, and the change curve of loss value is shown in Figure 19.

From Figure 13 and Figure 14, it can be observed that the accuracy of arc fault detection and the change in loss value exhibit stability, with no overfitting and underfitting. The constructed model demonstrates excellent performance. On the training set, the model shows rapid convergence with a significant increase in detection accuracy and a rapid decrease in the loss value. In the seventh iteration, the training accuracy reaches over 90%, the loss value decreases to below 0.4, and both tend to stabilize. On the validation set, due to the initial complexity of learned feature information, the convergence is slower for the first four iterations. However, after four iterations, the convergence speed increases, and the loss value decreases rapidly. At the 10th iteration, the validation accuracy reaches over 90%, the loss value decreases to below 0.3, and the change curve gradually stabilizes. Finally, on the training set, the detection accuracy reaches 99.72%, and the loss value decreases to 0.0801. On the validation set, the detection accuracy reaches 99.42%, and the loss value decreases to 0.0836.

### 5.2. Test Result Validation and Visualization

The characteristic data set of arc fault detection is constructed by assigning labels and thermal coding to the features after data processing. The composition of the data set is shown in Table 3.

To avoid one-sidedness and randomness in the detection results of a single validation set, a cross-validation method is adopted to prove the effectiveness of the detection results. The training and validation sets are redivided into a ratio of 3:1. One set of data is used as the validation set, while the other three sets of data are used as the training set. The process is repeated four times, and the results are shown in Table 4.

A total of 1000 sets of new samples are randomly selected as the test set to test the model and verify its generalization ability. The highest detection accuracy under different load states reaches 100%, and the lowest detection accuracy reaches 96.80%, exceeding 95%. Among the 1000 sets of test samples, 993 sets test correctly, and 7 sets test incorrectly. The detection accuracy reaches 99.30%. The detection effect is good, and the model has strong generalization ability. The following figure visualizes the detection results of the Attention-DRSN model in the form of a confusion matrix.

**Figure 20:**
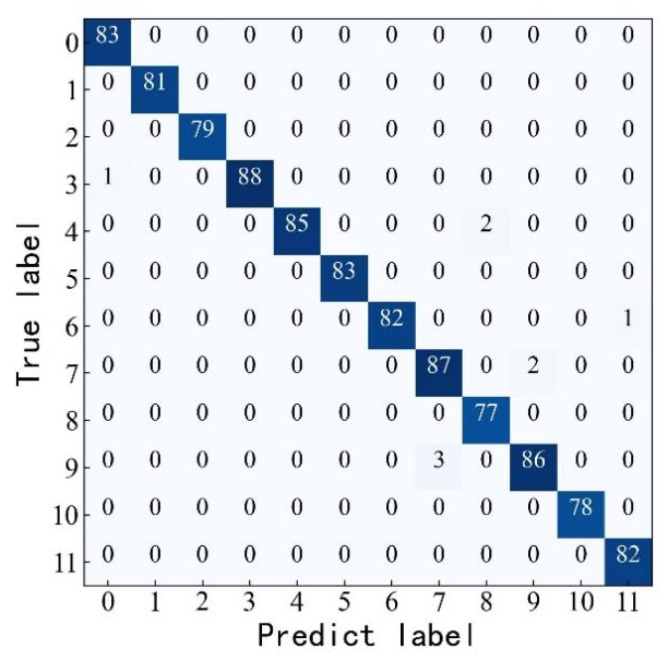
Confusion matrix for classification results.

As shown in Figure 20, among the 89 computer load arc fault samples, 3 samples are recognized as induction cooker load faults. Among the 89 induction cooker load arc faults samples, 2 samples are recognized as computer load faults. This is due to the fact that both induction cooker load and computer load fault current waveforms contain a large number of pulses and spikes, and the time–frequency image features extracted using the continuous wavelet transform are similar.

### 5.3. Comparison with Detection Methods Based on Single-Modal Feature

**Figure 21:**
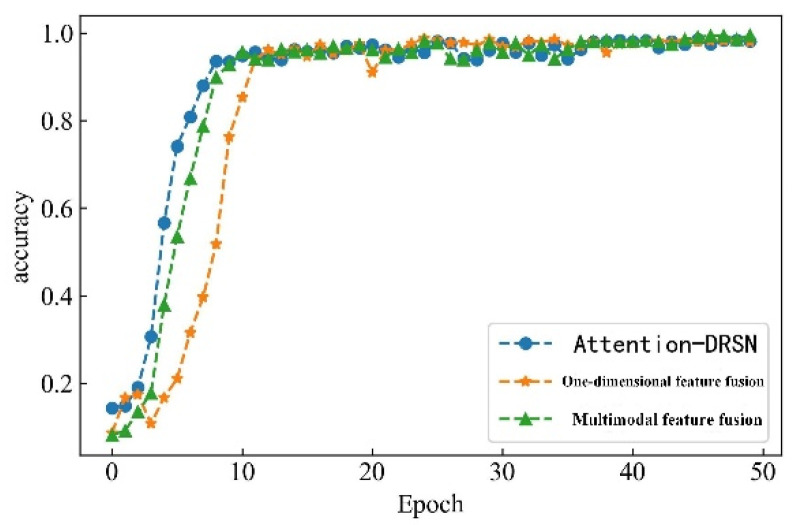
Accuracy change curve.

**Figure 22:**
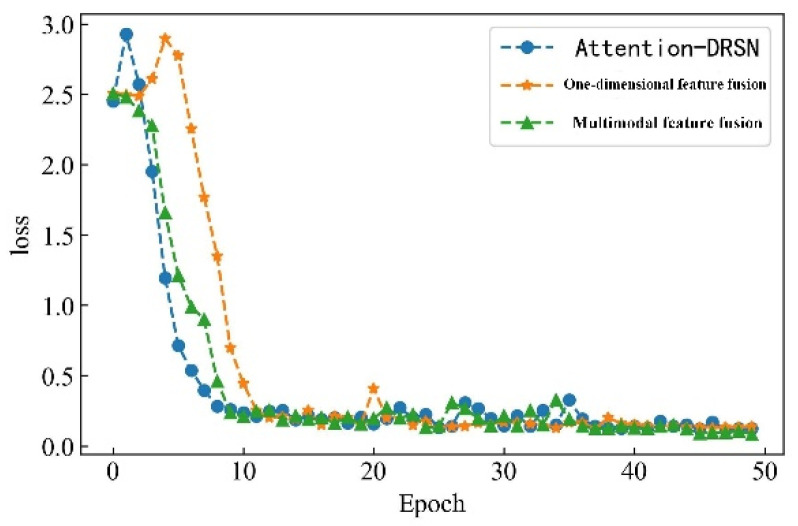
Loss value change curve.

To verify the superiority of multimodal features for series arc fault detection, this study constructs two arc fault detection models based on single-modal features, which are the Attention-DRSN model and the one-dimensional feature fusion model. The Attention-DRSN model utilizes the attention mechanism and the deep residual shrinkage network to detect arc faults from time–frequency image features. The one-dimensional feature fusion model uses the time-domain feature, frequency-domain feature, and wavelet packet energy feature. A comparative analysis is conducted with the proposed detection algorithm. The detection accuracies of the three methods on the validation set are illustrated in Figure 21, and the variation curves of the loss values are shown in Figure 22. All three methods achieve satisfactory results. Among them, the multimodal feature fusion method exhibits the best performance, with a detection accuracy of 99.33%. The detection accuracy of the Attention-DRSN model and the one-dimensional feature fusion method are quite similar, with accuracies of 98.11% and 98.04%, respectively.

The Attention-DRSN model demonstrates the fastest convergence rate among the three methods, the convergence rate of the multimodal feature fusion method model is second, and the convergence rate of the one-dimensional feature fusion model is the slowest. This is because the Attention-DRSN model only detects arc fault through time–frequency image features. The feature discrimination is high and the computer vision technology is relatively mature, which has excellent performance for image processing. Compared with the one-dimensional feature fusion mode, multimodal feature fusion can learn more effective information, and feature discrimination is more obvious, so the convergence rate is faster and the detection accuracy is higher.

### 5.4. Comparison with Other Published Detection Methods

In this paper, Python 3.8 is used for programming, and TensorFlow 2.4 is used to construct and train the neural network model. The method proposed in this paper is compared with the methods in the latest literature, and the results are listed in Table 5.

CHU Ruobo [17] extracted high-dimensional features of arc images by constructing a multilayer convolutional neural network. The abstract feature extraction of the convolutional neural network algorithm on faulty arc data is visualized in the form of time-domain gray-value images. Both algorithms in this study and in [17] achieve more than 95% accuracy in recognizing series arcs, and both can recognize load-type pairs. However, ref. [17] utilizes frequency-domain components to generate gray-value images for recognition, while this paper mainly uses time-domain and frequency-domain feature degree division for recognition, so the recognition effect and accuracy are better.

## 6. Conclusions

According to the idea of ensemble learning and decision-level feature fusion, an arc fault detection method based on multimodal feature fusion is proposed, and the following conclusions are obtained:

(1) Different modal features have different feature information, and the discrimination of feature data can be improved through feature fusion.

(2) Using different machine learning algorithms to process feature data can improve the quality of feature data and avoid interference from invalid and redundant information on detection results.

(3) The arc fault detection model based on ensemble learning and decision-level feature fusion can effectively solve the fusion problem of different modal features. Each submodel is independent and has stronger robustness.

(4) Compared with the arc fault detection method based on single-mode features, the detection method based on multimodal feature fusion has higher detection accuracy and better performance, with an average detection accuracy rate of 98.87%, and the detection accuracy rate reaches 99.30% in 1000 groups of random samples

## Figures and Tables

**Table 1:** Experimental load type.

Serial Number	Load Type	Experimental Load	Power Rating
1	Linear	Incandescent lamps	100 W
2	Linear	Incandescent series inductor	100 W
3	Linear	Electric hair dryer	400 W
4	Nonlinear	Induction cooker	1200 W
5	Nonlinear	Computer	350 W
6	Nonlinear	Hand drill	500 W

**Table 2:** Selection of scaling.

Serial Number	Experimental Load	Scaling Parameter *a*
1	Incandescent lamps	64
2	Incandescent series inductor	64
3	Electric hair dryer	64
4	Induction cooker	64
5	Computer	64
6	Hand drill	32

**Table 3:** Dataset composition.

Experimental Load	Load Status	Original Sample	Expanded Sample	Label	Thermal Coding
Incandescent lamp	Normal	62	530	0	[1 0 0 0 0 0 0 0 0 0 0 0]
	Arc fault	62	530	1	[0 1 0 0 0 0 0 0 0 0 0 0]
Series inductance of incandescent lamps	Normal	62	530	2	[0 0 1 0 0 0 0 0 0 0 0 0]
	Arc fault	62	530	3	[0 0 0 1 0 0 0 0 0 0 0 0]
Hair dryer	Normal	62	530	4	[0 0 0 0 1 0 0 0 0 0 0 0]
	Arc fault	62	530	5	[0 0 0 0 0 1 0 0 0 0 0 0]
Induction cooker	Normal	62	530	6	[0 0 0 0 0 0 1 0 0 0 0 0]
	Arc fault	62	530	7	[0 0 0 0 0 0 0 1 0 0 0 0]
Computer	Normal	62	530	8	[0 0 0 0 0 0 0 0 1 0 0 0]
	Arc fault	62	530	9	[0 0 0 0 0 0 0 0 0 1 0 0]
Electric hand drill	Normal	62	530	10	[0 0 0 0 0 0 0 0 0 0 1 0]
	Arc fault	62	530	11	[0 0 0 0 0 0 0 0 0 0 0 1]

**Table 4:** Cross-validation.

Serial Number	Loss Value	Accuracy Rate (%)
1	0.1199	98.53
2	0.0962	99.26
3	0.1041	98.47
4	0.0973	99.15
Mean	0.1043	98.87

**Table 5:** Comparison with other published detection methods.

Paper	Detection Method	Detection Accuracy	Experiment Load	Computational Complexity (Highest Order)
This paper	Attention-DRSN	98.87%	6	Linear and nonlinear
Reference [17]	TDV-CNN	97.7%	5	Linear
Reference [27]	IEWT-ELM	97.85%	7	Mixed load
Reference [28]	Deep auto-encoding network	98.56%	8	Nonlinear
Reference [29]	SVM	88.33%	3	Nonlinear

## Data Availability

Not applicable.
